# Preoperative Fall Risk Assessment Score as a Prognostic Factor in Esophageal Cancer Patients after Esophagectomy

**DOI:** 10.3390/jcm10245966

**Published:** 2021-12-19

**Authors:** Keita Kouzu, Hironori Tsujimoto, Yusuke Ishibashi, Hanae Shinada, Isawo Oikawa, Yoji Kishi, Nariyoshi Shinomiya, Hideki Ueno

**Affiliations:** 1 Department of Surgery, National Defense Medical College, Saitama 359-0042, Japan; dj27qd_t01312kk@yahoo.co.jp (K.K.); yuusuke0102_0908@yahoo.co.jp (Y.I.); hanae_shinada@yahoo.co.jp (H.S.); isa0922_artstrt@yahoo.co.jp (I.O.); ykishi-3su@ndmc.ac.jp (Y.K.); ueno_surg1@ndmc.ac.jp (H.U.); 2 Department of Integrative Physiology and Bio-Nano Medicine, Saitama 359-0042, Japan; shinomi@ndmc.ac.jp

**Keywords:** accidental falls, frailty, esophageal cancer, prognosis, risk assessment

## Abstract

The current study investigated the impact of preoperative fall risk assessment score (FRAS) on long-term prognoses in patients with esophageal cancer (EC). A total of 161 patients with EC who underwent curative surgery were classified into a high-risk (95, 41.0%) and low-risk (66, 41.0%) groups according to their FRAS. This study investigated the relationships between the FRAS and clinicopathological findings and prognoses. Accordingly, patients in the high-risk group were significantly older and had a significantly higher Charlson comorbidity index than those in the low-risk group. No significant difference was found in pathological findings between both groups. The high-risk group had significantly lower overall survival (OS) and relapse-free survival (RFS) rates than the low-risk group (*p* = 0.004 and 0.001, respectively). Multivariate analysis identified high FRAS as an independent prognostic factor for poor OS, with a hazard ratio of 1.75 (*p* = 0.033). Moreover, re-analysis of the data after excluding age as a category showed that the high-risk group had significantly worse OS (*p* = 0.004) and RFS (*p* = 0.003) than the low-risk group. The FRAS can, therefore, be considered a useful method for assessing frailty and a potential prognostic factor for EC.

## 1. Introduction

Frailty is a multidimensional clinical condition characterized by physiological decline, such as loss of physical ability, metabolic function, and cognition [1,2,3]. Given the increase in life expectancy, with the consequent increase in the number of elderly individuals, the significance of frailty in various diseases has gained attention. Studies in the field of oncology have reported that more than half of older patients with cancer exhibit pre-frailty or frailty [4]. Moreover, mounting evidence has found that frailty is associated with postoperative complications, adverse events of chemotherapy, and long-term prognosis in gastrointestinal cancer [5,6,7,8]. Generally, clinical trials involving cancer treatment that form the basis of guidelines tend to be conducted on relatively young patients, such as those under 70 years of age, with no consensus on surgery or chemotherapy for the elderly having yet been established. However, elderly patients deemed “fit” could tolerate intense treatment similar to young patients, suggesting that some people previously excluded from intense treatment owing to their advanced age may have been able to receive the same treatment as younger patients with proper evaluation. Thus, frailty assessment is particularly important in the selection of cancer treatment. Although no gold standard screening tool for frailty has yet been available, several methods for assessing frailty have been developed [9,10,11,12,13,14]. However, only limited numbers of simple and objective evaluation tools exist.

We had previously reported that the fall risk assessment score (FRAS), an objective and useful method of evaluating frailty, was associated with long-term prognosis following gastrectomy for gastric cancer [15]. FRAS has recently been used in several hospitals to assess patient-specific risk for falls during hospitalization from the perspective of medical safety management [16]. Several FRASs have been available and usually consist of items such as age, medical history, physical dysfunction, activity status, mental impairment, medications, and assistance required for toileting. Given that previously studies have found an association between falls and frailty, especially balance and gait [17,18], the FRAS may potentially reflect the frailty of the patient.

Esophageal cancer (EC) is the sixth most common cause of cancer-related death worldwide [19]. Given that the esophagus lacks a serous membrane, patients with EC are prone to progression, necessitating intensive treatments, such as esophagectomy and chemoradiation, for advanced disease. EC has been strongly linked to frailty not only because it promotes poor nutrition, as with gastric cancer, but also because it easily causes obstruction. The present study has been the first to investigate the relationship between FRAS and long-term prognosis among patients with EC.

## 2. Materials and Methods

### 2.1. Patients

This study retrospectively analyzed the medical records of 161 consecutive patients (134 males and 27 females; mean age, 69.8 ± 8.1 years old) who underwent radical esophagectomy for EC at the National Defense Medical College in Saitama, Japan, between January 2009 and December 2017. The median follow-up duration was 32.3 ± 26.7 months. All esophagectomy procedures were performed under thoracoscopy with artificial pneumothorax and two-lung ventilation.

Patients’ clinical records at admission for esophagectomy and pathological records were retrospectively evaluated for age, sex, body mass index, preoperative respiratory function test such as % vital capacity and forced expiratory volume 1.0%, age-adjusted Charlson comorbidity index (CCI), neutrophil-to-lymphocyte ratio (NLR), modified Glasgow prognostic score (GPS), American Society of Anesthesiologists Performance Status (ASA-PS), presence or absence of sarcopenia, histology, tumor size, tumor depth, lymph node metastasis, and pathological cancer stage. Sarcopenia was herein defined according to the psoas muscle index (PMI, cm^2^/m^2^), which was calculated from preoperative computed tomography images at the third lumbar vertebrae level and square of body height. Sex-specific cutoff values for low PMI, indicating sarcopenia, were set at the 25th percentile (3.86 and 2.87 cm^2^/m^2^ for males and females, respectively). Patients’ clinical data and pathological findings of EC recorded in accordance with the TNM classification system (version 8) of the Union for International Cancer Control were retrospectively evaluated.

### 2.2. Assessment of Fall Risk

Staff nurses assessed all patients for fall risk upon admission to the hospital for esophagectomy or for preoperative treatment in patients who received preoperative treatment. The fall risk assessment sheet of our hospital automatically returns the individual FRAS as previously reported (Table 1) [15]. The fall risk assessment includes seven categories: age, history of falls or syncope, physical dysfunction, activity status, mental dysfunction, medicines, and toileting needs. Altogether, these categories contain 46 fall risk items assessed individually. Risk scores were determined based on the following categories: two points for age ≥70; four points for a history of either falls or syncope and any number of mental dysfunctions; three points for each physical dysfunction or issue noted in the activity status; and one point for every checked item related to medicines and assistance required for toileting. “Accessories” (drip, gastric tube, and drain) or “wearing slippers and sandals” were not provided points in this study given that these fall risk items were not related to frailty as previously reported. Nurses determined the individual fall risk and implemented appropriate countermeasures based on patient interviews, assessment, and the calculated FRAS. The average FRAS of the enrolled patients was 4.8 ± 3.26 points, with a median of 4 points (range 0–15 points). Patients were divided into two groups according to their FRAS, with a cutoff value of 4 points indicating a high risk for falls: high-risk group (FRAS ≥ 4, 95 patients, 59.0%) and low-risk group (FRAS score < 4, 66 patients, 41.0%) (Table 2).

### 2.3. Perioperative Management

All patients received an oncologically appropriate plan according to the guidelines for EC treatment as described below [20,21]. Either neoadjuvant chemotherapy (NAC; 80 mg/m^2^ of cisplatin and 800 mg/m^2^ of 5-fluorourasil) or neoadjuvant chemoradiotherapy (NACRT; 70 mg/m^2^ of cisplatin and 700 mg/m^2^ of 5-fluorourasil plus 41.4 Gy of radiation) was provided for patients with clinical stage II or III disease before undergoing esophagectomy. Among the 111 patients with pathological stage II and III disease, 49 received NAC or NACRT. None of the patients enrolled herein received postoperative chemotherapy because adjuvant chemotherapy is not recommended owing to the lack of evidence of clinical benefit [22].

### 2.4. Statistical Analysis

All statistical analyses were performed using the JMP^®^ Pro software package version 14.3.0 (SAS Institute Inc., Cary, NC, USA). The Mann–Whitney U test and Pearson’s chi-square test were performed as appropriate. Survival rates were obtained using the Kaplan–Meier method, while statistical significance was determined using the log-rank test. To assess the relationship between the FRAS and clinicopathological parameters and long-term outcomes, univariate and multivariate analyses were performed using the Cox proportional hazards regression model. Data were expressed as mean ± standard deviation, with a *p* value of <0.05 indicating statistical significance.

## 3. Results

### 3.1. Fall Risk Assessment Score

**Figure 1:**
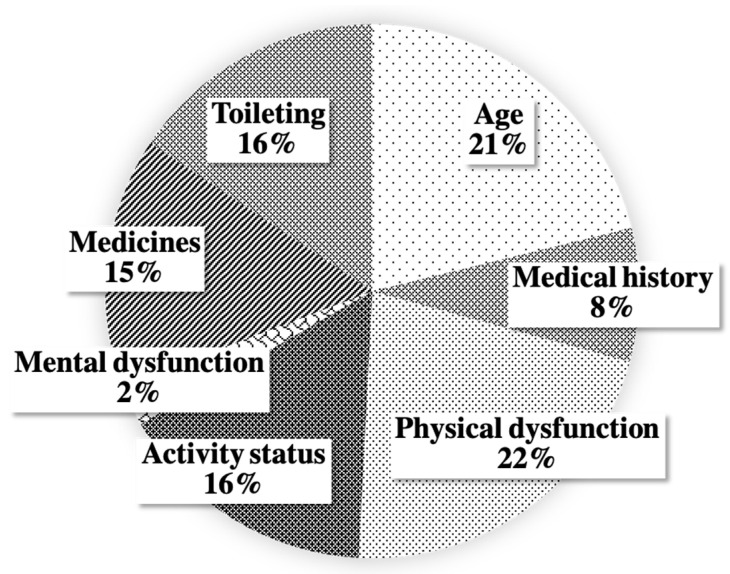
Percentage contribution of each category to the total fall risk assessment score (FRAS).

Table 1 shows the number of cases for each fall risk item of the FRAS. Accordingly, 84 patients (52.2%) were aged 70 years or older, while 14 (8.7%) had a history of falls. “Physical dysfunction” was found to contribute the most to the total score (171 points, 22%; Figure 1). The most frequently observed risk item was “going to the toilet at night”. The most frequently used medications were antihypertensives and diuretics.

The high-risk group was significantly older and had significantly higher CCI and ASA-PS than the low-risk group (Table 2). No significant differences in sex, body weight, body mass index, preoperative respiratory function, NLR, modified GPS, and presence or absence of sarcopenia were observed between the high- and low-risk groups. The mean PMI values were 4.80 and 3.36 for males and females, respectively. No significant differences in pathological findings, such as histology, tumor size, tumor depth, lymph node metastasis, and pathological stage, were found between the two groups.

### 3.2. Fall Risk Assessment Score and Esophageal Cancer Survival Rate

**Figure 2:**
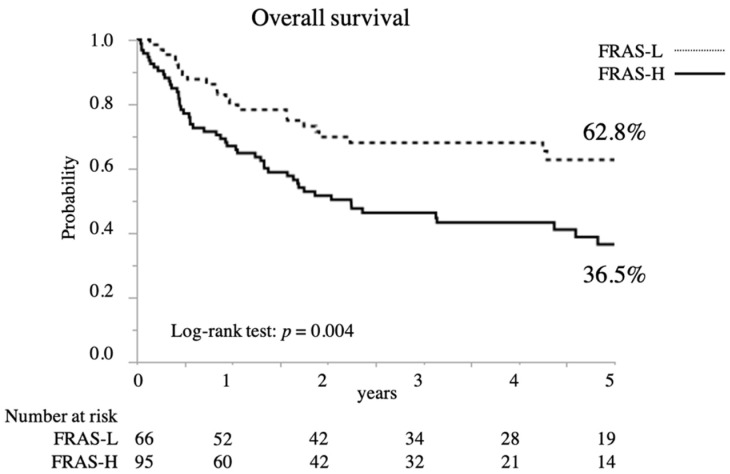
Overall survival in patients with esophageal cancer (EC) (*n* = 161) according to their FRAS.

**Figure 3:**
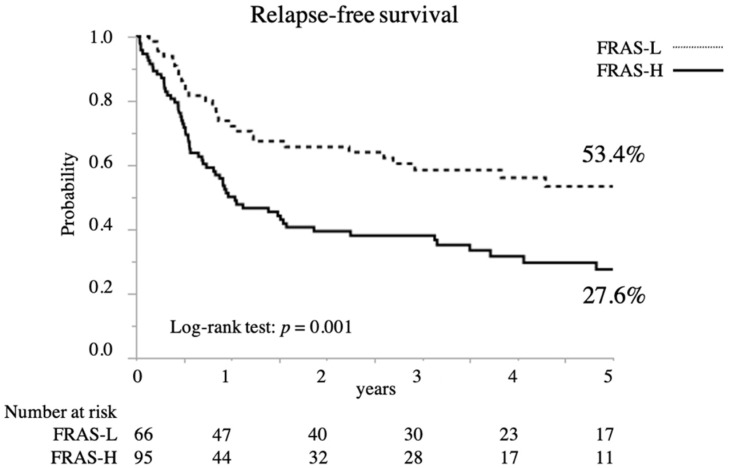
Relapse-free survival in patients with EC (*n* = 161) according to their FRAS.

Recurrence rates were 40.0% and 30.3% in the high- and low-risk groups, respectively, with no significant differences therein (Table 1). However, the high-risk group had a significantly lower overall survival (OS) rate (5-year OS 36.5% vs. 62.8%; *p* = 0.004; Figure 2) and relapse-free survival (RFS) rate than the low-risk group (5-year RFS 27.6% vs. 53.4%; *p* = 0.001; Figure 3).

Table 3 presents the results for univariate and multivariate analyses for OS. Univariate analysis demonstrated that the high-risk FRAS group and CCI ≥6 were significantly associated with OS. Meanwhile, multivariate analysis revealed that only the high-risk FRAS group (hazard ratio (HR): 1.75, 95% confidence interval (CI): 1.05–2.92; *p* = 0.033) was an independent prognostic factor for unfavorable OS. Similarly, only the high-risk FRAS group (HR: 1.89, 95% CI: 1.17–3.04; *p* = 0.009) was an independent prognostic factor for poor RFS (Table 4). Moreover, re-analysis of the data after excluding age as a category showed that the high-risk group had a significantly worse OS (*p* = 0.004) and RFS (*p* = 0.003) than the low-risk group (data not shown).

In addition, univariate analysis was performed to evaluate which factors in the FRAS affect long-term prognosis, using the seven categories of the FRAS as analysis factors (Table 5). As a result, medical history (HR: 2.71, 95% CI: 1.51–4.86; *p* < 0.001) and activity status (HR: 2.44, 95% CI: 1.16–5.13; *p* = 0.018) were selected as prognostic factors for OS.

## 4. Discussion

Several studies have recently investigated the association between long-term prognosis and frailty in gastrointestinal cancers [5,23]. An important concept that could explain the association between frailty and cancer is cachexia, a condition in which complex metabolic abnormalities, such as those caused by malignancy, promote skeletal muscle mass and weight loss. Reports have shown that 80% of the patients with advanced cancers have cachexia and that 20% of cancer deaths can be attributed thereto [24]. In addition, it has become clear that immune and humoral factors involved in cancer progression are also involved in the formation of cachexia [25,26]. Understanding the degree of frailty among patients with cancer may improve our understanding of cancer progression.

The current study highlights the usefulness of FRAS as a measure of frailty. Our findings showed that high FRAS was significantly correlated with poor OS and RFS following esophagectomy for EC, with multivariate analysis subsequently identifying high FRAS as an independent factor for unfavorable prognosis. The FRAS is a non-invasive method for evaluating vulnerability in patients and a potentially useful prognostic indicator for EC. These results are consistent with those printed in a previous study on patients with gastric cancer [15].

Previous reports have suggested an association between fall risk and frailty considering that it reflects physical weakness [9,17,27,28]. However, the current study found no association between FRAS and sarcopenia. One of the reasons for this may be that all patients included herein had a low PMI, with most of them having sarcopenia. The present study used the 25th percentile as the cutoff value for sarcopenia; however, when sarcopenia was determined using previously reported cutoff values for sarcopenia in patients undergoing liver transplant (i.e., 6.36 and 3.92 cm^2^/m^2^ for males and females, respectively), 83.3% of our patients already had sarcopenia [29]. Particularly, determining the prognosis of EC based on the presence or absence of sarcopenia may be difficult given that patients with EC are often in a state of cachexia, have poor nutritional intake, and suffer from other conditions that can easily promote muscle weakness. Moreover, the definition of sarcopenia is based solely on physical findings, whereas frailty comprehensively evaluates the condition of the entire body and is thought to identify worse body conditions that are otherwise overlooked by sarcopenia alone. In fact, the “activity status” category, which is considered to be closely related to sarcopenia, contributed only 16% to the total FRAS of all patients, as shown in Figure 1. Considering that patients must have the ability to decide on their treatment, only a small number of patients with the “mental dysfunction” category were included. However, the other categories contributed equally to the total FRAS. This result suggests that the FRAS evaluates patients across multiple dimensions. On the contrary, given that the FRAS includes “age” as one of its categories and that the high-risk group included many elderly patients, the FRAS could have possibly contributed to the prognosis. The high number of patients with higher CCI in the high-risk group also supports this suspicion. However, the high-risk group had significantly poorer prognosis even after excluding age from the FRAS criteria, suggesting that the FRAS remains useful regardless of age. On the other hand, only medical history and activity status were selected as prognostic factors when univariate analysis was performed with each factor of FRAS. However, patients with mental dysfunction, for example, may not have been included in this group because sometimes they were unable to make the decision to undergo surgery, which may have affected patient selection bias.

The current study identified the FRAS as an independent prognostic factor for poor OS and RFS. Although the poor OS in the high-risk group can easily be explained by their older age and higher CCI, the same group also exhibited poor RFS despite no significant differences in pathological factors between the high- and low-risk groups. These results are consistent with previous reports showing that frailty was associated with poor long-term prognosis of cancer. These results are consistent with previous reports showing that frailty was associated with poor long-term prognosis in various cancers [4,30]. Some possible reasons include a negative spiral of malnutrition, weight loss, and fragility in the high-risk group, which may predispose them to cachexia and lower their resistance to chemotherapy after recurrence [25]. Cancer treatments and cancer bearing itself are significant stressors that can lead to frailty, which is an important factor to consider when balancing the risks and benefits of treatment.

Some studies have reported on the effectiveness of combined exercise and nutritional interventions for cancer patients with frailty [31,32]. Quist et al. found that an exercise training program improved not only muscle strength, but also the secondary outcomes of social well-being, anxiety, and depression in patients with unresectable advanced lung cancer [32]. It is not easy to implement these interventions simultaneously with treatment, as it requires multidisciplinary involvement. However, the secondary outcomes in the report are very important in terms of improving vulnerability characterized by a comprehensive sense of physiological decline, including loss of physical ability, metabolic function, and cognition. Thus, we speculated that the combination of exercise and nutritional intervention may improve long-term prognosis in FRAS high-risk patients.

This study has several potential limitations. First, this was a retrospective single-center study that included a relatively small number of patients. We expect to validate our findings in a prospective cohort. Second, this study examined the FRAS used in our hospital. In addition, intra- or inter-rater reliability in the assessment of FRAS could not be assessed in this study because we conducted a retrospective study. Our results need to be validated using a widely utilized assessment tool including intra- or inter-rater reliability. Finally, this study did not assess bone fragility, considering the physical dysfunction category. For example, bone densitometry and vitamin D information should be considered in a validation study in the future.

## 5. Conclusions

In conclusion, the current study showed that, among patients with EC, those who had a high FRAS exhibited poorer prognosis compared with those with low FRAS. Moreover, the FRAS is a useful method for assessing fall risk while simultaneously reflecting the degree of frailty and may be a prognostic factor in EC.

## Figures and Tables

**Table 1:** Fall risk assessment sheet with number of applicable cases in this study.

Category	Fall Risk Items	Score	No. of Cases
Age	70 years old or older	2	84
Medical history	History of fall	4	14
	History of syncope/convulsions/weakness attacks		2
Physical dysfunction	Visual impairment	3	14
	Hearing impairment		4
	Pain		9
	Dizziness		3
	Paralysis		3
	Numbness		7
	Anemia		2
	Bone and joint abnormalities (contracture, deformation, and so on)		2
	Muscle weakness		4
	Wobble		7
	Sudden progress		0
	Others		2
Activity status	Using wheelchair, cane, walker	3	10
	Need assistance when moving		4
	Abnormal posture		1
	Rest for more than 3 days (bedridden)		0
	Accessories (drip, gastric tube, drain)		13
	Wearing slippers and sandals		14
	Others		0
Mental dysfunction	Disorientation	4	1
	Unconsciousness		0
	Dementia		0
	Decreased judgment, understanding, and attention		4
	Depression		0
	Restlessness (hyperactivity, wandering)		0
	Others		0
Medicines	Opioid	1	1
	Psychotropic drugs		3
	Sleeping pills		15
	Painkillers		8
	Hypoglycemic agent		14
	Anti-parkinsonian drugs		1
	Enema/laxative		13
	Antihypertensive/diuretic		43
	Chemotherapeutic drugs		5
	Antiplatelets and anticoagulants		12
	Others		5
Toileting	Assistance required for toileting	1	2
	Frequent urination		3
	Urine and fecal incontinence		1
	Urethral catheter placement		2
	Going to the toilet at night		115
	Others		1

**Table 2:** Patients’ clinicopathological factors.

	FRAS High-Risk	FRAS Low-Risk	Total	*p*-Value
	(*n* = 95, 59.0%)	(*n* = 66, 41.0%)	(*n* = 161)	
Age	71.9 ± 7.7	66.8 ± 7.8	69.8 ± 8.1	<0.001 *
Sex male/female	78/17	56/10	134/27	0.647
Body weight (kg)	54.4 ± 11.5	56.3 ± 10.1	55.2 ± 11.0	0.449
Body mass index (kg/m^2^)	20.7 ± 3.8	21.1 ± 3.3	20.8 ± 3.6	0.671
%vital capacity	102.4 ± 18.8	101.3 ± 15.2	102.0 ± 17.4	0.642
Forced expiratory volume 1.0%	2.3 ± 0.6	2.5 ± 0.7	2.4 ± 0.6	0.26
CCI score	5.1 ± 1.7	3.8 ± 1.5	4.6 ± 1.7	<0.001 *
Neutro lymph ratio	3.1 ± 2.4	2.6 ± 1.5	2.9 ± 2.1	0.371
Modified GPS	0.7 ± 0.6	0.8 ± 0.5	0.8 ± 0.5	0.658
ASA-PS	2.4 ± 0.5	2.2 ± 0.4	2.4 ± 0.5	0.024 *
Sarcopenia	18 (18.9%)	9 (13.6%)	27 (16.8%)	0.375
Histology SCC/others	87/8	58/8	145/16	0.44
Tumor size (mm)	48.5 ± 28.8	49.2 ± 29.7	48.8 ± 29.0	0.904
Tumor depth T3 ≤ (Yes/No)	50/45	27/39	77/84	0.143
Lymph node metastasis N2 ≤ (Yes/No)	34/61	21/45	55/106	0.601
Pathological cancer stage 3 ≤ (Yes/No)	41/54	27/39	68/93	0.291
NAC or NACRT	27 (38.7%)	22 (51.2%)	49 (44.1%)	0.236
Follow-up period (months)	69.1 ± 26.1	38.9 ± 26.4	32.3 ± 26.7	0.004 *
Recurrence	38 (40.0%)	20 (30.3%)	58 (36.0%)	0.208

FRAS: fall risk assessment score, CCI: age-adjusted Charlson comorbidity index, GPS: Glasgow prognostic score, ASA-PS; American Society of Anesthesiologists Performance status, SCC: squamous cell carcinoma, NAC: neoadjuvant chemotherapy, NACRT; neoadjuvant chemoradiotherapy. Asterisks for significance values. Data are expressed as the mean ± standard deviation.

**Table 3:** Prognostic factors for the overall survival.

		Univariate Analysis			Multivariate Analysis		
		HR	95% CI	*p-*Value	HR	95% CI	*p-*Value
Age	≥70	1.5	0.95–2.38	0.08	1.11	0.67–1.84	0.683
Sex	Male	1.4	0.72–2.72	0.321	1.23	0.63–2.41	0.55
Sarcopenia	Yes	1.16	0.64–2.11	0.622	1.18	0.64–2.20	0.59
ASA-PS	3	1.48	0.94–2.31	0.089	1.12	0.67–1.87	0.672
CCI	≥6	1.95	1.22–3.10	0.005 *	1.48	0.84–2.61	0.174
Fall risk assessment score	High-risk	2.02	1.25–3.29	0.004 *	1.75	1.05–2.92	0.033 *

HR: hazard ratio, CI: confidence interval, ASA; American Society of Anesthesiologists Performance status, CCI: age-adjusted Charlson comorbidity index. Asterisks for significance values.

**Table 4:** Prognostic factors for the relapse-free survival.

		Univariate Analysis			Multivariate Analysis		
		HR	95% CI	*p-*Value	HR	95% CI	*p-*Value
Age	≥70	1.43	0.93–2.19	0.099	1.1	0.69–1.75	0.683
Sex	Male	1.48	0.79–2.79	0.221	1.36	0.72–2.59	0.344
Sarcopenia	Yes	1.05	0.60–1.83	0.868	1.05	0.59–1.86	0.861
ASA-PS	3	1.37	0.90–2.09	0.142	1.08	0.67–1.75	0.75
CCI	≥6	1.66	1.07–2.57	0.024 *	1.23	0.72–2.10	0.447
Fall risk assessment score	High-risk	2.09	1.34–3.27	0.001 *	1.89	1.17–3.04	0.009 *

HR: hazard ratio, CI: confidence interval, ASA; American Society of Anesthesiologists Performance status, CCI: age-adjusted Charlson comorbidity index. Asterisks for significance values.

**Table 5:** Prognostic factors included in FRAS for the overall survival.

		Univariate Analysis		
		HR	95% CI	*p-*Value
Age	Yes	1.54	0.98–2.43	0.061
Medical history	Yes	2.71	1.51–4.86	<0.001 *
Physical dysfunction	Yes	1.45	0.88–2.40	0.143
Activity status	Yes	2.44	1.16–5.13	0.018 *
Mental dysfunction	Yes	1.2	0.44–3.29	0.724
Medicines	Yes	1.14	0.73–1.78	0.565
Toileting	Yes	1.1	0.68–1.80	0.689

HR: hazard ratio, CI: confidence interval. Asterisks for significance values.

## Data Availability

The data that support the findings of this study are available from the corresponding author upon reasonable request.
